# Neotectonics of the Sea of Galilee (northeast Israel): implication for geodynamics and seismicity along the Dead Sea Fault system

**DOI:** 10.1038/s41598-020-67930-6

**Published:** 2020-07-20

**Authors:** Luca Gasperini, Michael Lazar, Adriano Mazzini, Matteo Lupi, Antoine Haddad, Christian Hensen, Mark Schmidt, Antonio Caracausi, Marco Ligi, Alina Polonia

**Affiliations:** 1 Istituto di Scienze Marine, ISMAR, CNR, Geologia Marina Bologna, Bologna, Italy; 2 Department of Marine Geosciences, University of Haifa, Haifa, Israel; 3 Centre for Earth Evolution and Dynamics, University of Oslo, Oslo, Norway; 4 Department of Earth Sciences, University of Geneva, Geneva, Switzerland; 5 GEOMAR Helmholtz Centre for Ocean Research Kiel, Kiel, Germany; 6 Istituto Nazionale di Geofisica e Vulcanologia, INGV, Palermo, Italy

**Keywords:** Solid Earth sciences, Geochemistry, Geodynamics, Geology, Geophysics, Seismology, Tectonics

## Abstract

The Sea of Galilee in northeast Israel is a freshwater lake filling a morphological depression along the Dead Sea Fault. It is located in a tectonically complex area, where a N-S main fault system intersects secondary fault patterns non-univocally interpreted by previous reconstructions. A set of multiscale geophysical, geochemical and seismological data, reprocessed or newly collected, was analysed to unravel the interplay between shallow tectonic deformations and geodynamic processes. The result is a neotectonic map highlighting major seismogenic faults in a key region at the boundary between the Africa/Sinai and Arabian plates. Most active seismogenic displacement occurs along NNW-SSE oriented transtensional faults. This results in a left-lateral bifurcation of the Dead Sea Fault forming a rhomb-shaped depression we named the *Capharnaum Trough*, located off-track relative to the alleged principal deformation zone. Low-magnitude (M_L_ = 3–4) epicentres accurately located during a recent seismic sequence are aligned along this feature, whose activity, depth and regional importance is supported by geophysical and geochemical evidence. This case study, involving a multiscale/multidisciplinary approach, may serve as a reference for similar geodynamic settings in the world, where unravelling geometric and kinematic complexities is challenging but fundamental for reliable earthquake hazard assessments.

## Introduction

Strike-slip deformation zones are complex tectonic domains generally showing high lateral variability. This is due mainly to strain partitioning, which can develop transtensive and transpressive deformations in response to local crustal heterogeneities or to changes in the regional stress field. Fault bends and oversteps create zones of diffuse deformation, which could mask the tracks of main active fault segments. On the other hand, diachronicity in fault activation-deactivation often creates complex patterns recording multiphase tectonic processes. Another uncertainty is whether or not deformations observed at the surface are expression of deep-seated tectonic structures. Where lakes or inland seas develop, particularly in tectonically subsiding areas, waterborne seismic reflection surveys can be a powerful tool to overcome these problems, because the relatively homogeneous and continuous sedimentary sequence which fills the depression may enhance imaging of tectonic structures, facilitating kinematic reconstructions. In the subaqueous environment, the good coupling between seismic source, substratum and receivers, together with a relative simplicity in field operations, permits acquisition of densely spaced grids of high-resolution subsurface images. Where deformation rates are high relative to sediment supply, exposed tectonic lineaments may be detected by combining seismic reflection profiles and high-resolution morpho-bathymetric maps. However, in order to produce reliable maps of active faults, these data should be coupled with other information, such as local seismicity, major historical earthquakes, geodetic velocity fields, as well as geochemical evidence of tectonically triggered fluid flow.

**Figure 1:**
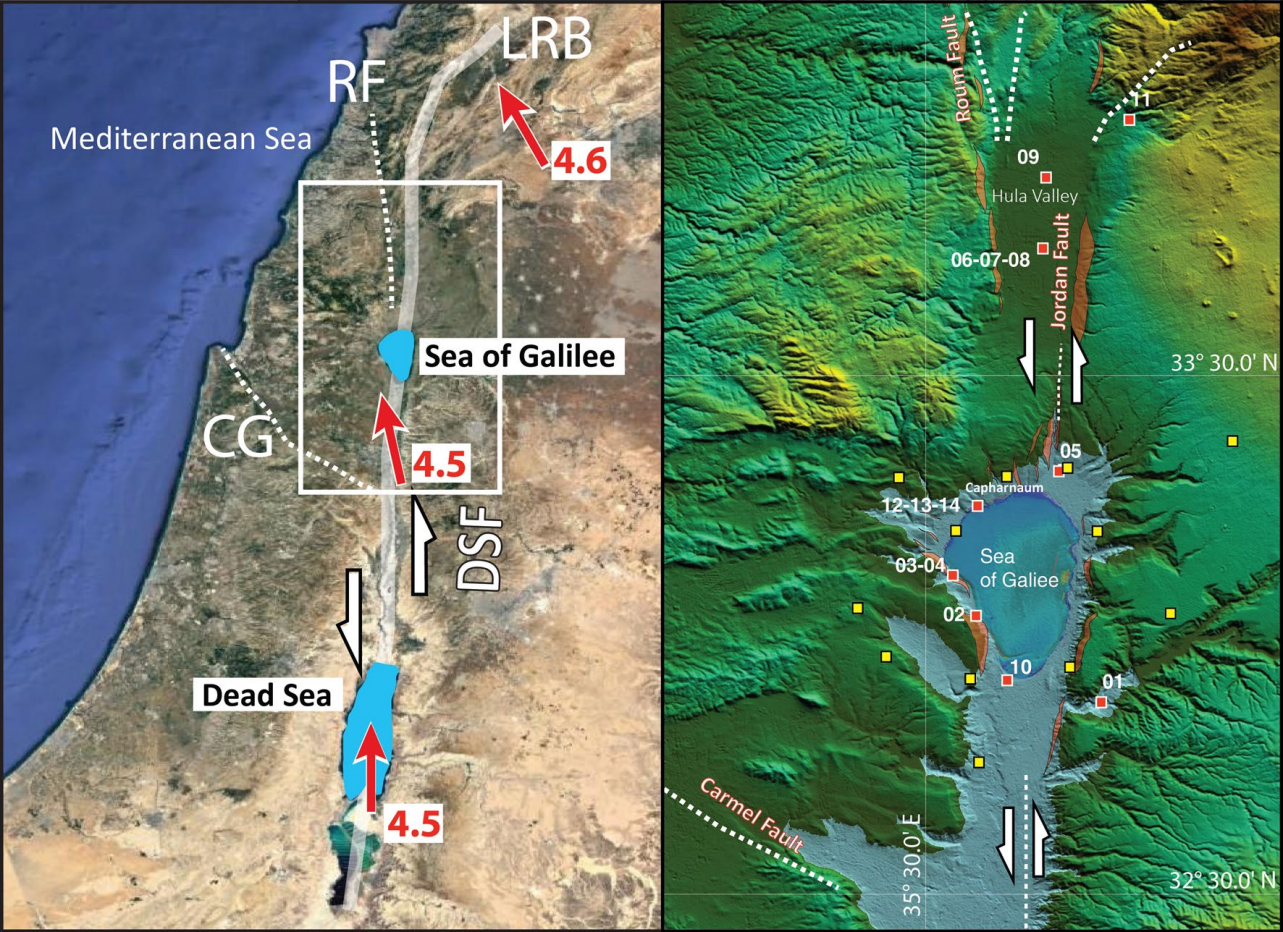
Left: Google Earth image (https://www.google.it/intl/it/earth/; Map data: Google, Data SIO, NOAA, U.S.: Navy, NGA, GEBCO, Image Landsat/Copernicus) of the Dead Sea Fault schematically illustrating the plate tectonic setting of the study area; DSF = Dead Sea Fault; CF = Carmel Fault; RF = Roum Fault; LRB = Lebanon Restraining Bend; red arrows and numbers show the direction and amount of movement of the Arabian Plate with respect to the Africa Plate in mm per year, from Wdowinski et al.^61^. Right: SRTM-plus topography (https://search.earthdata.nasa.gov/search/) highlighting main structural features in northern Israel (white rectangle on the left); dashed white lines mark the major tectonic features; red boxes with numbers indicate location of fluid sampling stations; yellow boxes indicate location of seismograph stations; topographic scarps of probable tectonic origin are marked by a red transparent pattern (image editing, Adobe Illustrator CS6).

In this study, we analyse a multidisciplinary/multiscale dataset collected in and around the Sea of Galilee (SoG) in northeast Israel (Fig. 1), along the Dead Sea Fault (DSF) system. It included newly-acquired or reprocessed seismic reflection profiles (single- and multi-channel), morpho-bathymetric (echo soundings and side-scan sonar) images, geochemical and seismological data, collected in the frame of an international project in 2018^1^. Our work focused on reconstructing the neotectonics of this complex region, that was struck in the past by destructive earthquakes, with the aim to improve geodynamic interpretation and seismic hazard assessment.

## Background

### Geological setting

The SoG or Lake Kinneret (Kinnor = harp in Hebrew) in northeast Israel, is a freshwater lake with a maximum extent of 12 × 20 km in the E-W and N-S directions, respectively. Its surface is over 200 m below mean sea level, has a maximum water depth of ~ 40 m, and is filled by a sedimentary sequence reaching a thickness of over 6 km^2^. The SoG basin is one of a series of rhomb-shaped grabens developing along the DSF system^3^, a continental transform fault displacing laterally the Africa/Sinai and Arabian plates at a rate of about 4.5 mm/year (Fig. 1). Tectonic deformations in this region are mainly oriented N-S, except for the Carmel-Gilboa fault (CG) oriented SE-NW, and a major eastward bending of the DSF main track towards Lebanon (LBR), to the south and north of the SoG, respectively (Fig. 1).

Imaging of active subsurface faults in the SoG is challenging due to the presence of gas in the shallow sedimentary section, which absorbs the high-resolution seismic energy^4–6^. Some faulted, tilted and flexed sediments interpreted as indications for relatively recent tectonic activity were observed along the margins of the basin and in the lake’s depocenter^7–9^. Additional indirect evidence for active tectonics was inferred by earthquake surface ruptures detected in the vicinity of the SoG^10,11^ as well as by the high heat-flow measurements^12^.

The origin and evolution of the SoG tectonic depression have been broadly debated. Numerous studies suggest that the it began to form in the Neogene^13–15^ as a result of left lateral strike–slip motion along the N-S oriented plate boundary. However, Rosenthal et al.^16^ suggested that the lake’s depression is not a pull-apart basin, but formed as a result of Pleistocene subsidence caused by salt withdrawal and normal faulting. Multi-channel seismic data from the lake led Hurwitz et al.^8^ to conclude that the basin is continuous at depth, while Reznikov et al.^9^ indicated a number of faults that cut through the basement. Ben-Avraham et al.^2^ divided the SoG into two distinct structural units, the first comprising a southern sub-basin and most of the present-day lake as a pull-apart basin extending southwards. Since the deepest part of the basin is located well south of the deepest bathymetric depression, it was assumed that the latter is an actively subsiding young feature^8^. The northern sector of the basin has been suggested to be an asymmetrical (to the E) half-graben, due to rotational opening along the DSF system^2^, while other studies point to a N-S trending marginal fault along most of the western margin^8,9,17^. Based on magnetic data, Schattner et al.^18^ proposed that a single fault only is present in the lake (aside from the eastern boundary fault), which cuts it from SSW to NNE. Deformation along the CG Fault system (Fig. 1) is also not completely understood. Combined GPS and levelling measurements estimated a right-lateral slip rate of 3.5 mm/year^19^. This is in contrast with observations of Hofstetter et al.^20^, who described left-lateral fault mechanism solutions for local seismicity. GPS measurements carried out during several years range from a left-lateral rate of 0.9 ± 1.1 mm/year^21,22^, to a right-lateral rate of 4.5 mm/year^23^, or to a similar rate but opposite in polarity (left-lateral) of 4 mm/year^24^. Although these rates are significantly higher than those found by geological observations, according to Nof et al.^25^, they cannot be ignored. Thus, the debate continues.

**Figure 2:**
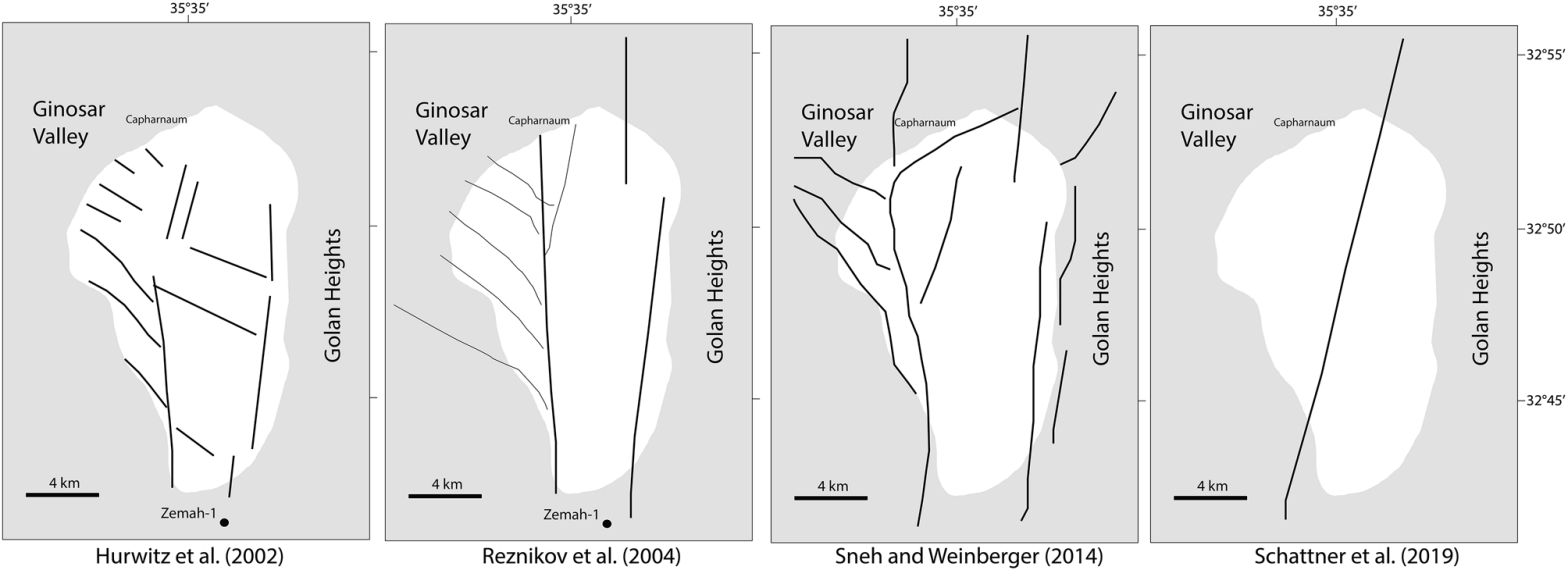
Different tectonic models proposed by various authors for the Sea of Galilee (image editing, Adobe Illustrator CS6).

These difficulties in defining a univocal model for deformation in the SoG (Fig. 2) and along the CG Fault, reflect the uncertainties on the nature and activity of potentially seismogenic faults in this wrench tectonic domains characterized by relatively low deformation rates, a general problem also typical of other geotectonic settings.

### Historical earthquakes

Large magnitude destructive earthquakes have struck the SoG region throughout historical times, as reported by paleoseismological and archeoseismological studies^26^. The estimated recurrence interval of Mw = 6 earthquakes, of the order of 10^2^ years, decreases to 10^3^ years for Mw ~ 7 earthquakes^27,28^. Historical earthquake catalogues report strong damage during the years 303, 363, 551, 749, 1,202, 1759, and 1837 AD^29^. The earthquake of January 18th, 749 was a large-magnitude event, with extensive damage in the northern part of the Jordan valley, the destruction of Tiberias and Beit-She’an, and surface ruptures on the western shore of the SoG^30^. The earthquake of May 20, 1,202 is probably the strongest affecting this region in historical times^29^. The earthquake of January 1st, 1837 caused severe damage in Tiberias and Safed, again along the western shore of the SoG. Reports on this earthquake^31^ are particularly accurate, and include description of seiches affecting the lake, as well as largest macroseismic intensities located west of the SoG and the DSF principal displacement zone.

## Results and discussion

### Seismological observations

**Figure 3:**
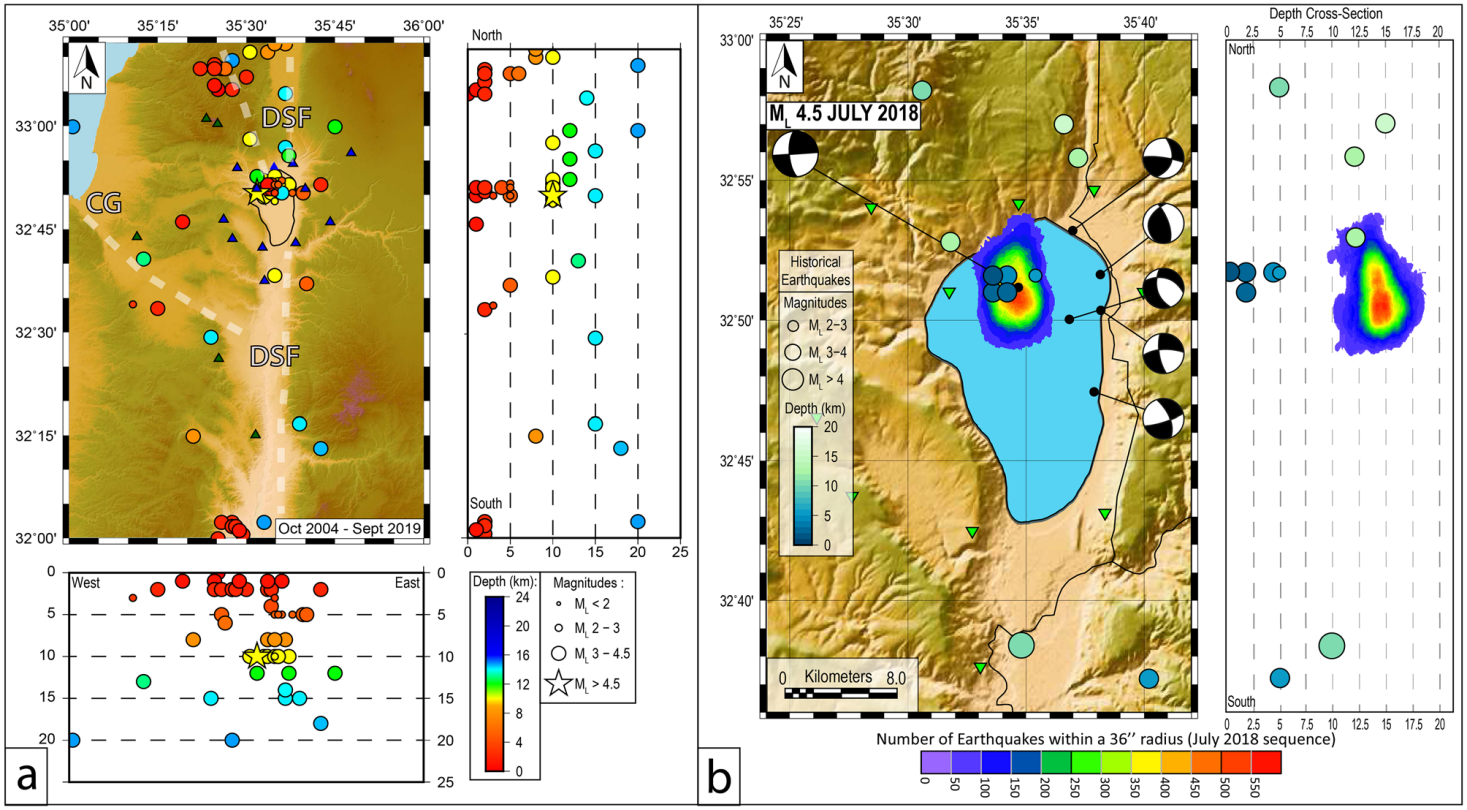
Earthquake distribution in Israel and surrounding regions. (**a**) Events recorded from the 1st of January 2000 to present (from Israeli Seismological Service. (**b**) 4th of July 2018 seismic sequence. Black beach-balls along the eastern side of the SoG are from ref.^32^, while focal mechanism of the main July 2018 shock is from current study. Topography data are from https://search.earthdata.nasa.gov/search/. Maps compiled using the GMT package (https://www.generic-mapping-tools.org/); image editing using Adobe Illustrator CS6.

Seismicity affecting Israel and the surrounding regions in recent times (2000–2017) has been moderate (Mw < 5) and distributed between three deformation zones along the DSF system (Fig. 3a): -the main DSF track, running approximatively N-S; -a secondary branch running NW along the trace of the CG Fault; -a third NNW oriented segment bifurcating from the main DSF track north of the SoG. The main DSF track is site of aligned strike-slip focal mechanisms along the SoG western shore^32^. While the CG Fault zone is characterized by diffuse seismicity, the NNW-striking deformation zone to the north of SoG is marked by clusters of seismic events (Fig. 3b), and appears the most seismically active region in terms of both number and magnitude of events. Instrumental seismic activity around the lake over the last decades indicates a seismogenic depth reaching down to 20 km^32^, with shallower events recorded close to the northern shore (Fig. 3b).

The October 2013 seismic sequence, with magnitudes up to M_l_ = 3.7 earthquakes took place along this NNW aligned structure (https://www.gii.co.il/news/earthquakes-jul2018) and was followed by a similar sequence on the 4th of July 2018, during our campaign^1^. The mainshock of this latter sequence was an M_L_4.6 earthquake followed by four M_L_ > 4.0 aftershocks between 12 and 15 km of depth. The earthquake density colour distribution map of Fig. 3b highlights the location of the main rupture, as well as the orientation of the seismic pattern. We note that the sequence is well confined, bounded by a sharp eastern limit corresponding to the DSF principal displacement zone, and developing mostly in the NW sector of the lake (Fig. 3b). The temporal evolution of the 2018 sequence suggests a main rupture at about 15 km of depth, subsequently migrating upwards with smaller magnitudes and larger lateral dispersal. The entire sequence lasted approximately one month, and its kinematic indicates NNW–SSE left-lateral strike slip displacement.

### Geophysical data

**Figure 4:**
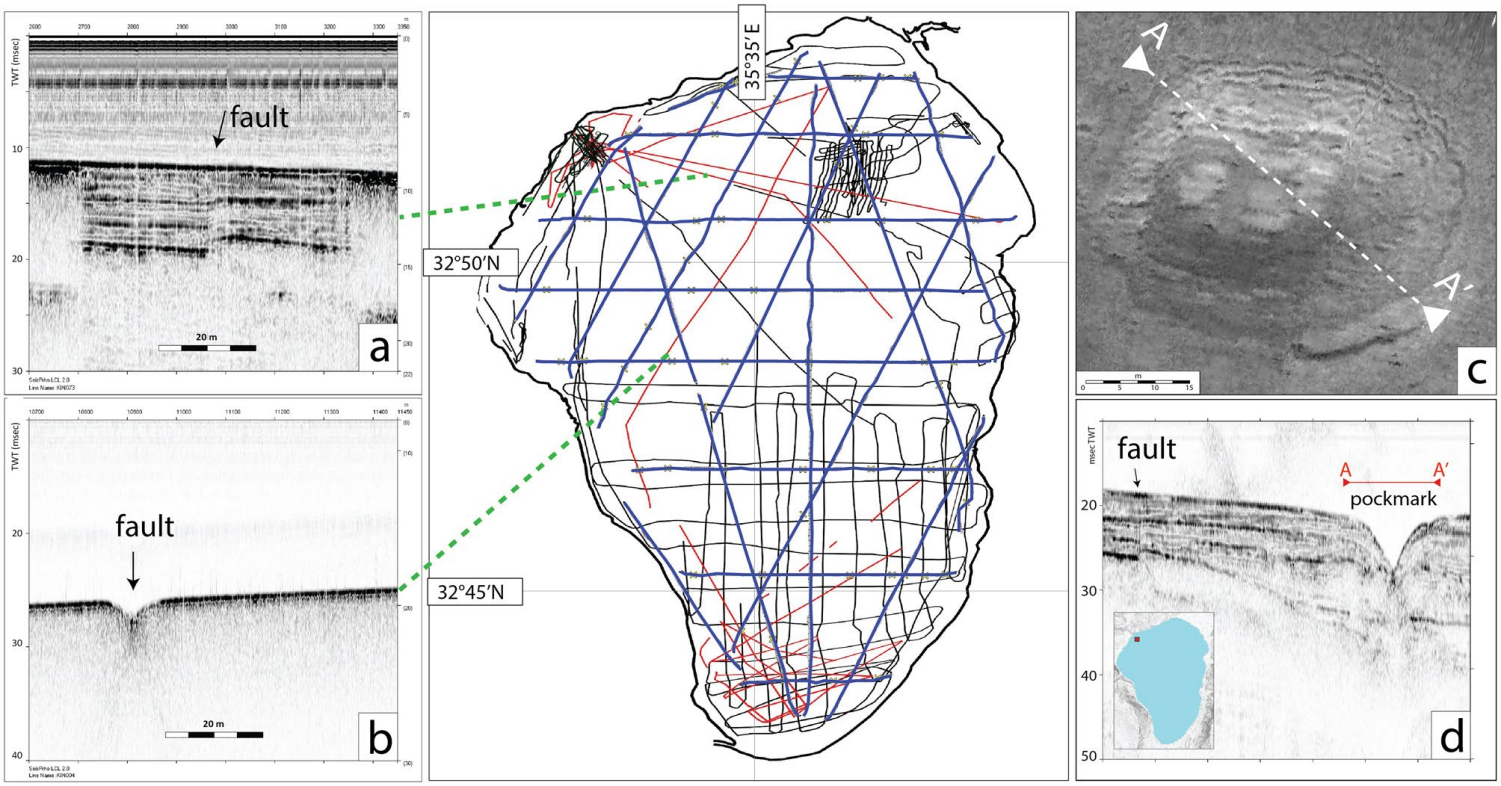
Seismic reflection profiles collected during several decades in the Sea of Galilee. Red lines: 2017 survey; black lines: single channel profiles; blue lines: MCS survey. (**a**, **b**, **d**) Chirp-sonar profiles collected during the 2017 campaign from the SoG showing different expressions of active faults (arrows). (**a**, **d**) Gas-free areas, with sub-vertical displacements of the sediments reaching up to the lake floor. (**b**) No-penetration, with displacement marked by a morphological notch. (**c**) Pockmark side-scan sonar image in the NW sector of the SoG (section view in **d**); these features, ranging in size from a few meters to some tens of meters in diameter, are located close to active faults. Red box indicates location of the lake-floor backscatter image. Map compiled using the GMT package (https://www.generic-mapping-tools.org/); image editing using Adobe Illustrator CS6.

To map tectonic deformations affecting the SoG floor and its subsurface, we used a set of geophysical data including morpho-bathymetric maps, side-scan sonar images, high resolution chirp-sonar profiles, as well as multichannel seismic reflection lines acquired in 1997 and completely reprocessed to obtain depth-migrated sections. Coverage of the analysed geophysical dataset is displayed in Fig. 4.

The majority of available high-resolution chirp-sonar seismic reflection profiles revealed no acoustic penetration due to the widespread presence of gas in the sediments^6^. However, two sectors of the lake close to the SW and NW shores allowed good penetration of the seismic energy, up to a few tens of meters. Thus, chirp sonar data were used to detect active faults visible as sediment displacements in areas free of gas (Fig. 4a–c), or as small notches where gas in the sediments hampered penetration of the signal below the lake floor (Fig. 4b). The enhanced penetration of the signal in sectors with active faults was interpreted as due to the effect of seismically triggered gas-release occurring preferentially along these structures (e.g.,^6^ and references therein). This mechanism could also have caused topographic depressions and pockmarks observed in backscatter images from the lake floor (Fig. 4c). Side-scan sonar backscatter images were only partially useful in detecting fault traces, although this technique has been shown to highlight focused earthquake-related ruptures after major seismic events^33^. This might suggest that the floor of the SoG is not affected by recent (tens of years) coseismic ruptures. Nevertheless, in areas such as the NW sector, several mound-shaped structures and pockmarks, ranging in size from a few meters to tens of meters, were visible in the vicinity of fault traces. Similar features have been observed in other wrench tectonic domains, related to gas and fluid expulsion^34–36^.

**Figure 5:**
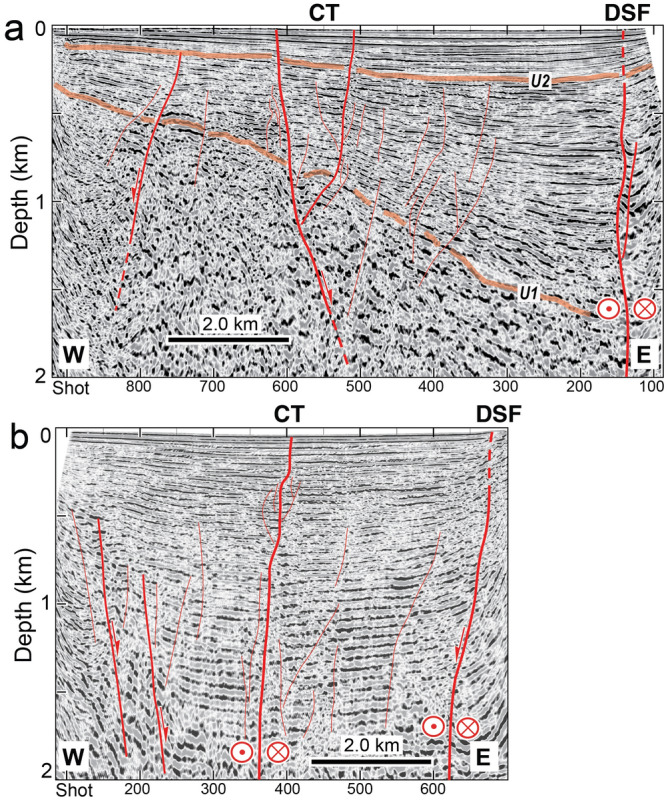
Interpreted depth-migrated multichannel seismic reflection (MCS) profiles KIN_05 (**a**) and KIN_07 (**b**) crossing the SoG (location in Fig. 6). Seismic units marked by two major unconformities (U1 and U2) record different stages in the basin formation. Main DSF trace, as well as trastensional faults bounding the Capharnaum Trough (CT), affect the uppermost part of the sedimentary sequence reaching up to the lake floor (thick red lines). See Supplementary Figs. S2 and S3 for uninterpreted data. Image editing, Adobe Illustrator CS6.

**Figure 6:**
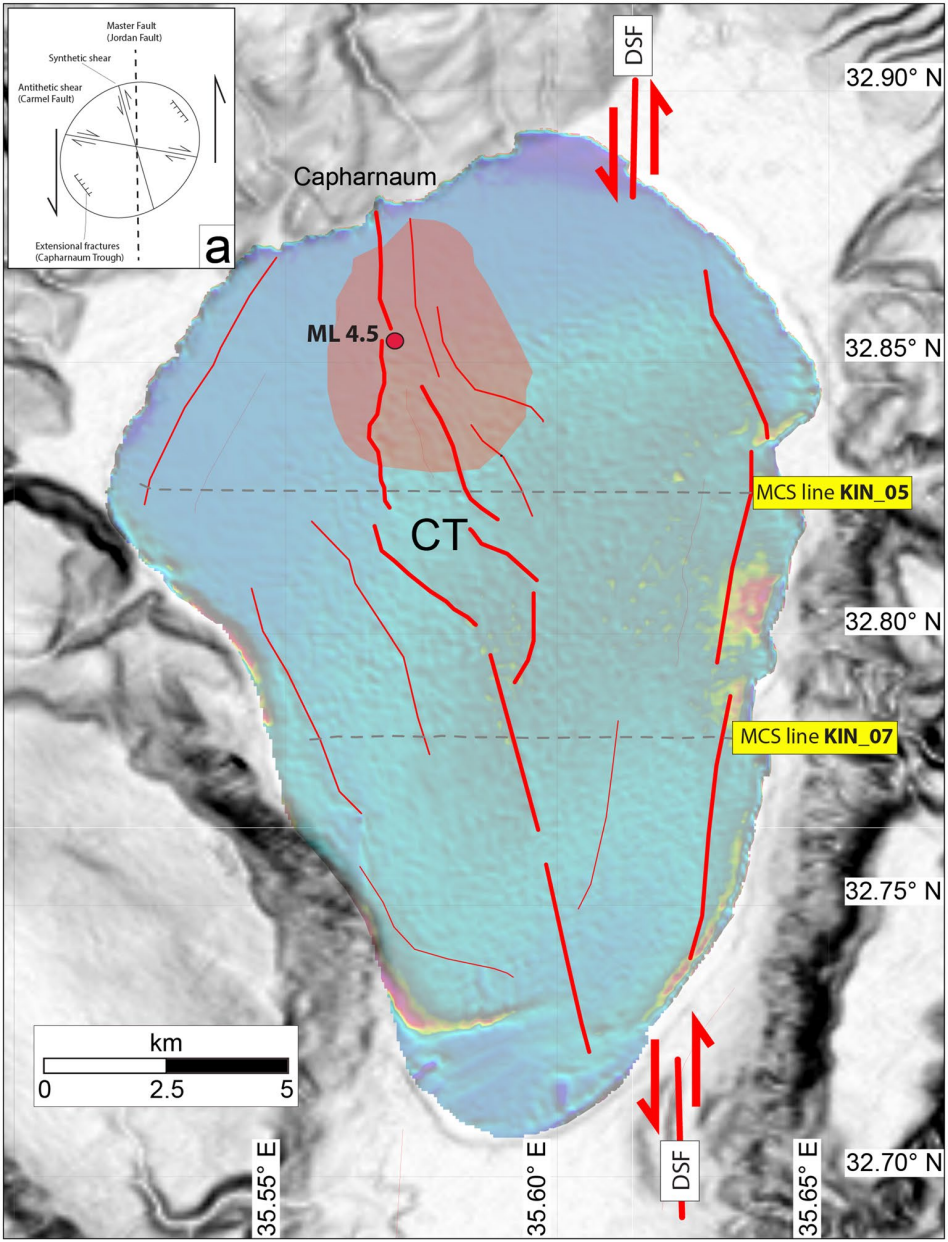
Neotectonic map of the Sea of Galilee compiled using available geophysical data; all faults (thick red lines) have a sinistral strike-slip component; main epicentres of the July 2018 earthquake sequence (> Mw 3.5) are indicated by blue circles, with yellow pattern boundings the 250–300 events isoline. Inset: orientation and relative motion of observed strain patterns in our study area in comparison with a reference strain ellipse. Topography onshore is from https://search.earthdata.nasa.gov/search/. Map compiled using the GMT package (https://www.generic-mapping-tools.org/); image editing using Adobe Illustrator CS6.

Multichannel seismic reflection (MCS) lines, reprocessed to fit the purpose of this work (see Methods), enabled detection of kinematic indicators within the sedimentary sequence. Figure 5 shows two E-W oriented profiles crossing the SoG in its central part (location in Fig. 6). The northernmost line KIN_05 (Fig. 5a) shows a thick wedge of well layered deposits whose thickness increases eastwards. A number of unconformities can be identified within the section, as also underlined by previous authors^8,9^. We focused on two major unconformities, U1 and U2 in Fig. 5a, which constitute the boundaries of three main seismostratigraphic units marking different phases in the basin evolution: from bottom to top, pre- (below U1), *syn*- (between U1 and U2) and *post-* (above U2) extension. This refers to the onset and subsequent deactivation of a main extensional phase of the DSF principal displacement zone along the eastern shore of the SoG, which created the asymmetric depression coinciding with the basin depocenter. *Syn*-rift sediments show fanning and growing structures, indicating tectonic subsidence driven by a dip-slip extensional (trans-tensional) fault(s) at the eastern edge of the basin (Fig. 5a). Stratigraphic correlations by Rezinkov et al.^9^ suggest that U2 should coincide with the earliest stage of SoG formation, 2.0–1.7 Ma (TCB in their study), while U1 could be related to a major change/rearrangement of the DSF system (PQ5 in^9^) not clearly constrained, but more recent than 1 Ma. In Line KIN_05 (Fig. 5a), sediments within these units appear affected by pervasive faulting and fracturing, although we recognize two main high-angle faults reaching up to the lake floor. While the strike-slip character of such faults cannot be recognized in the 2-D sections, their opposite dipping and internal geometries are compatible with the presence of a negative flower structure forming a tectonic depression.

The southernmost Line KIN_07 (Fig. 5b) crosses a narrower sector of the lake. Here, the negative flower structure and the fan-shaped geometries observed in Line KIN_05 merge into a single sub-vertical fault cutting through sub-horizontal reflectors, in place of the fan-shaped geometries. The 3-D expression of these structural patterns was constrained by analysing the entire dataset, leading to the compilation of the tectonic map displayed in Fig. 6, where major faults (thick red lines) reaching up to the lake’s floor should be considered active.

If we exclude the DSF principal deformation zone running along the eastern shore (Fig. 6), the most prominent feature observed in the seismic sections is the NNW-SSE transtensive structure forming at a left-lateral bifurcation propagating from the lake’s centre towards the village of Capharnaum (Fig. 1). For this reason, we have named this feature the *Capharnaum Trough* (CT). Interestingly, this suspended depression is shifted towards west relative to the lake’s depocenter and is controlled by a set of opposite verging transtensive faults. As further evidence of its activity, we note that the distribution of epicentres detected during the 2018 seismic sequence is centered on the northern extension of the CT (red area in Fig. 6).

NNW structural elements recognized in the SoG can be viewed as synthetic Riedel shears (strain ellipse in Fig. 6) aligned with local seismicity recorded during the 2018 event (Fig. 3). In this reconstruction, the SoG appears to have developed at a major bifurcation between two active branches of the DSF system: a main, N-S strand flanking the eastern shore of the lake, and the 347°-oriented CT, which diagonally crosses the centre of the lake. On the eastern side of the lake, the main DSF strand is expressed by a series of extensional-transtensional scarps at the base of the steepest topographic gradients, forming a cumulative left-lateral offset of about 2 km between the southern and the northern shores (Fig. 6).

### Geochemistry

**Figure 7:**
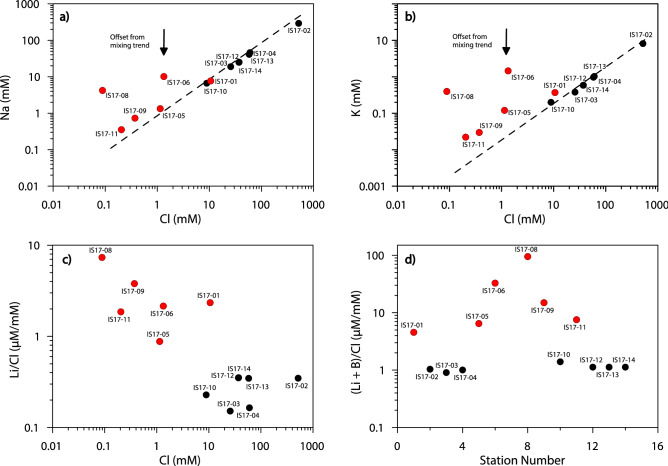
Pore water elements plots for Cl versus Na (**a**) and Cl versus K (**b**) show that the Group 1(Black symbols) generally show higher salinities compared to Group 2 (red symbols). Enrichments of fluid-mobile elements (B, Li) is observed for Group 2 stations (**c**,**d**). Diagrams were compiled using the GMT package (https://www.generic-mapping-tools.org/), while image editing was carried out using Adobe Illustrator CS6.

**Figure 8:**
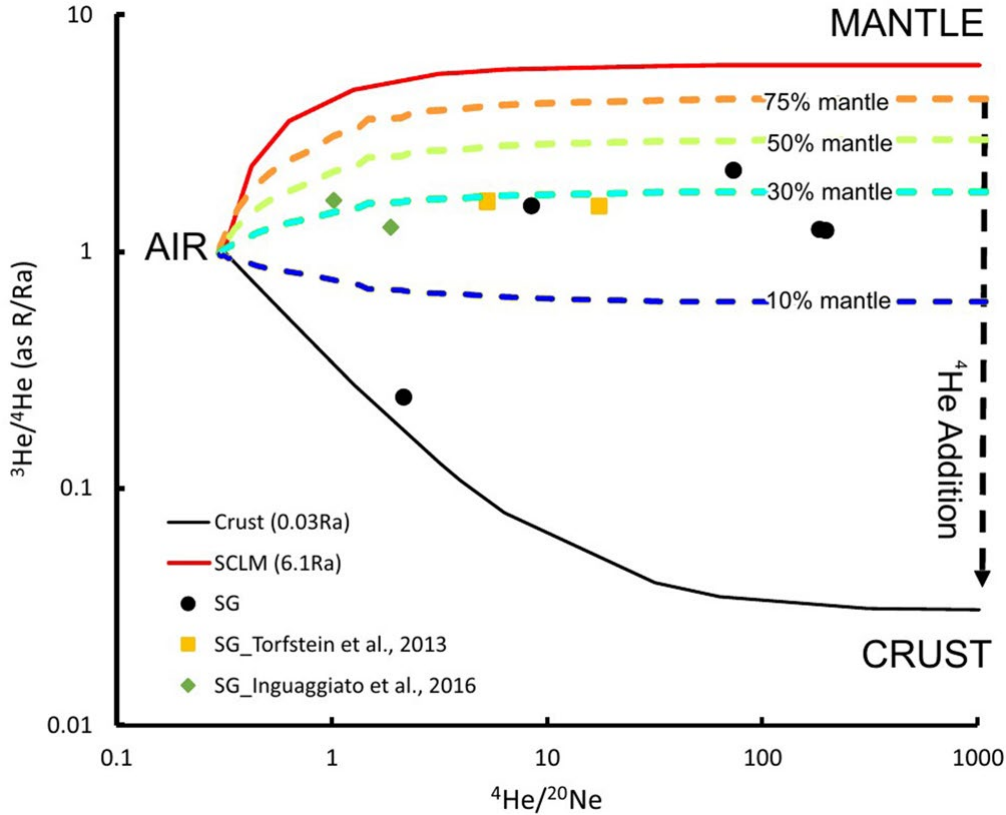
Plot of the measured He isotopes versus ^4^He/^20^Ne ratio showing the integrity of the He isotope results. The curves represent mixing between airsaturated water (1 Ra), sub-continental lithospheric mantle (SCLM, 6.1 Ra), and crust (0.03 RA). Diagrams compiled using the GMT package (https://www.generic-mapping-tools.org/); image editing using Adobe Illustrator CS6.

Fluids carried to the surface through migration pathways such as active faults, may provide insights into depth and pervasiveness of tectonic deformations. One of the objectives of our geochemical survey was to investigate whether deformations observed by geophysical data are related to deep-rooted structures. All geochemical results are presented in Tables 1, 2 and 3 and Figs. 7 and 8, and will be discussed in detail below.

**Table 1:** Summary of sampled stations and water composition analyses of the fluids divided in groups according to plots (Fig. 7)

Station no.	Group type	Locality	Lat. N	Long. E	T (°C)	pH	Conductivity mS/m	Comments	B	Mn	Ca	Na	Mg	Sr	Si	Ba	Li	K	Cl	Br	SO_4_	I
									(mM)	(µM)	(mM)	(mM)	(mM)	(µM)	(mM)	(nM)	(µM)	(mM)	(mM)	(µM)	(mM)	(µM)
IS17-01	1S	Hamat Gader	32° 40.982′	35° 40.038′	44.1	6.21	1.97	Large artificial pool with numerous bubbling points, smell of H_2_S, greenish microbial colonies growing.	0.023	0.027	4.060	7.678	1.779	55.914	0.425	987.02	24.799	0.370	10.595	58.806	1.343	0.375
IS17-02	1S	Hamat Tiberias	32° 46.032′	35° 32.997′	59.4	5.11	48,520	Natural spring with salt crusts and greenish-yellow microbial mats. Vigorous water stream and gas bubbling	0.356	1.524	85.292	291.932	26.276	742.807	0.619	#####	179.791	8.143	517.341	3044.238	8.027	3.836
IS17-03	1S	Fuliya	32° 48.385′	35° 31.665′	28	6.65	3.48	2x2 m pool, close to the beach and rare bubbles	0.019	0.000	4.694	18.892	2.988	21.294	0.347	642.24	3.885	0.380	25.668	69.772	1.192	0.040
IS17-04	1S	Fuliya	32° 48.395′	35° 31.680′	30.4	6.43	7.10	Spring on the beach close to IS17-03. The spring is partly covered by concrete. Through an aqueduct the water is flushed to the south in Jordan river. High flow rate	0.051	0.012	8.402	45.934	5.100	51.623	0.346	#####	9.918	1.023	60.247	180.757	2.735	0.202
IS17-05	2S	Bethsaida	32° 54.523′	35° 37.745′	~ 30			Fresh water spring in the Jordan park. No gas bubbling observed	0.006	0.011	1.539	1.334	1.299	2.770	0.498	#####	1.001	0.120	1.139	4.621	0.258	0.033
WIS17-06	2	Hula Valley N3-well	33° 7.352′	35° 36.793′	Mild			Irrigation well initially drilled up to 2500 m for hydrocarbon exploration, later plugged at 500 m depth. Water supposedly comes no deeper than 500 m but deeper migrating fluids cannot be excluded. This station sampled the upper tap of the well head expelling only cold water	0.036	0.838	4.987	9.054	7.767	17.246	1.191	#####	2.403	1.070	1.336	153.157	0.004	17.018
IS17-08	2	Hula Valley well	33° 7.422′	35° 36.773′	Mild			Well located 70 m NW of N3. The well is open and entirely covered by carbonate concretions. Seeping mild temperature water and gas bubbles. Depth of well ~ 50 m but uncertain. Smell of H_2_S.	0.005	1.368	12.430	3.794	10.461	13.487	0.893	#####	0.000	0.245	0.089	102.744	0.000	18.280
IS17-09	2	Hurshat. Tal1 -well	33° 11.515′	35° 36.979′				Well at Hurshat Tal locality (Tal1) drilled at depth of 878 m, T=21°C, discharge 1200 m^3^/h. Sampling was done from the large pipe flushing the water that ultimately reaches the lake	0.004	0.000	2.550	0.730	0.534	4.210	0.126	82.79	1.412	0.030	0.373	2.253	0.373	0.024
IS17-10		Sea of Galilee (Degania)	32° 42.576′	35° 34.756′				Lake water at shore at locality Degania, sampled during the morning	0.010	0.020	1.177	6.650	1.631	9.012	0.157	473.31	2.024	0.200	8.881	30.197	0.657	0.091
IS17-11	2S	Banias	33° 14.908′	35° 41.667′	Cold			Fresh water spring in the north of Israel. Used by Romans with ruins and temples. Cold temp, the spring is located at the base of the cliff likely following fault plane as migration pathway	0.001	0.001	1.769	0.353	0.488	2.935	0.140	61.26	0.380	0.022	0.204	0.000	0.480	0.000
IS17-12	1S	Tabgha	32° 52.350′	35° 33.053′	Mild			Inside S. Peter confraternity. Large pool. Not far from beach	0.029	0.002	7.202	25.088	2.989	67.944	0.299	620.26	13.115	0.588	37.237	158.028	1.070	0.000
IS17-13	1S	Tabgha	32° 52.379′	35° 33.005′	Mild			Inside Franciscan confraternity. Not far from beach. This large flushing pipe supposedly originates from a spring located in the Benedictine confraternity. Smell of H_2_S	0.045	0.081	9.784	41.049	4.280	99.607	0.311	641.58	20.102	0.973	58.033	240.884	1.744	0.153
IS17-14	1S	Tabgha	32° 52.375′	35° 33.021′	Mild			Inside Franciscan confraternity. Not far from beach. The pipe is an overflow of the pool where unique lobsters thrive inside. 100 m from IS17-12	0.029	0.000	7.030	24.858	2.968	66.848	0.318	608.48	12.949	0.586	36.952	156.679	1.062	0.000
IS17-14	1S	Tabgha	32° 52.375′	35° 33.021′	Mild			Duplicate	0.029	0.000	7.050	25.028	2.976	67.489	0.300	611.89	13.176	0.581				

S, spring site

The same station numbers are used in Tables 2 and 3.

**Table 2:** Major gas components of the sampled localities.

Station number	He (ppmV)	H_2_ (ppmV)	O_2_ (%-Vol.)	N_2_ (%-Vol.)	CH_4_ (ppmV)	C_2_H_6_ (ppmV)	C_3_H_8_ (ppmV)	CO (ppmV)	CO_2_ (%-Vol.)	H_2_S/SO_2_ (%-Vol.)	δ^13^C_CO2_ (‰ VPDB)	δ^13^C_CH4_ (‰ VPDB)	δ^13^C_C2H6_ (‰ VPDB)	δD_CH4_ (‰ VSMOW)
1S17-01	663	bdl	3.03	86.54	3,680			2.9	7.23		− 15.5	− 18.7		6
1S17-02	4,154	bdl	3.5	80.48	3,260			2.8	12.31		− 9	26.2		281
1S17-03	135	3.8	6.85	87.67	450			11	2.88		− 20			
IS17.03					0.9	bdl	bdl		2.95	0	− 21.9			
IS17.03					3.8	bdl	bdl		3.12	0	− 24.5			
IS17.03^a^					1.4	bdl	bdl		2.46	0	− 20.5			
IS17.04^a^					7	bdl	bdl		2.61	0	− 20.4			
IS17.05^a^					10.3	bdl	bdl		1	0	− 24.2			
IS17.06^a^					72,680	11	0		30.85	0	− 5.7	− 65.8	− 28.4	
IS17.06												− 65.7		
IS17.07					9E+05	34.7	0.7		9.05	1.47	− 5.3	− 66.5	− 26.4	
1S17-07	bdl	2.8	0.09	1		53		5	10.84		− 7	− 68.4		− 221
IS17.08					6E+05	10.1	8.4		32.52	1.57	− 5.9	− 58.7	− 29.7	
1S17-08	bdl	3.8	0.12	1.58				0.6	34.77		0.8	− 62.8		− 258
IS17.09^a^					1.6	bdl	bdl		1.26	1.84	− 16.6			
IS17.11^a^					1.2	bdl	bdl		0.66	3.85	− 20.9			
IS17.14^a^					1.8	bdl	bdl		0.87	5.33	− 20.6			
IS17-12-14^b^					1.11E^−02^			3.12 E^−05^	13.95		3.12E^−05^	13.95		

^a^Headspace gas sample, bdl = below detection limit. ^b^Inguaggiato et al.^42^, where units in are expressed in ccl^−1^.

**Table 3:** Noble gas composition for the sampled localities.

Station number	R/Ra	^4^He/^20^Ne	[^4^He] ppm	[^20^Ne] ppm	Rc/Ra	Err + /− Rc/Ra	^40^Ar ppm	^40^Ar + /− err	^40^Ar/^36^Ar
1S17-01	2.20	73.51	774.39	10.54	2.21	0.0177	10,578	5.542	302.8
1S17-02	1.24	185.72	1,690.35		1.24	0.0112			
1S17-02	1.22	197.90	1801.16	9.10	1.22	0.0094	12,697	4.176	309.2
1S17-03	1.56	8.54	140.83	16.50	1.58	0.0157	10,234	3.627	301.7
IS17-05^a^	1.62	5.33							
1S17-07		2.86	0.32	0.11			596	0.222	297.7
1S17-07	0.24	2.17	0.26	0.12	0.11	0.0043	908	0.229	293.1
1S17-08		2.93	0.26						
1S17-08		2.22	0.20	0.09			137	0.035	286.4
IS17-11^b^	1.64	1.03							
IS17-12-14^b^	1.26	1.89							
IS17-12-14^a^									

^a^Torfstein et al.^42^. ^b^Inguaggiato et al.^43^.

According to water geochemistry (Table 1), the stations sampled during this study (Fig. 1 for location) can be subdivided in two groups (Fig. 7). Group 1 (stations 2, 3, 4, 12, 13, 14) includes localities along the western and north western SoG shore, as well as Station 10 in the southwest. These samples generally show higher salinities and plot along a mixing line between freshwater and seawater (shown for Cl *vs.* Na and Cl *vs*. K; Fig. 7a, b). Stations of Group 2 (1, 5, 6, 8, 9, 11), sampled along the main strand of the DSF, show a slight offset with respect to conservative elements Na and K (Fig. 7a, b). Group 2 stations show relative enrichments of fluid-mobile elements (B, Li) (Fig. 7c, d). It is worth noting the enrichment of dissolved constituents at Hamat Tiberias (Station 2), where pervasive salt deposits due to the precipitation of solutes are present. Here, we observe the most anomalous values of our survey, with relative enrichment of at least one order of magnitude higher than other samples measured.

Gas geochemistry (Table 2) also suggests two groups of samples, partly overlapping with the observed water trends. Stations 1, 2, 3 are N_2_-dominated, with values ranging between 80.5 and 88%. CO_2_ at these sites ranges from 2.8 to 12.3%, with minor amounts of CH_4_ up to 0.37%; δ^13^C_CH4_ and δ^13^C_CO2_ range from − 18.7 to 26.2‰ and from − 9 to − 20.0‰, respectively. Distinctive values have been observed in the Hula Valley (Stations 6–7–8), where gas samples are methane dominated (up to 88%), with minor amounts of C_2+_ hydrocarbons and with CO_2_ as high as 35%. The δ^13^C_CH4_ and δ^13^C_CO2_ are from − 68.4 to − 58.7‰ and from − 7 to 0.8‰ respectively. Except for one station, with a particularly low R/Ra ratio of 0.24, the measured He isotopes ratios (Table 3) are higher than 1 (R/Ra span from 1.2 to 2.2). The ^4^He/^20^Ne ratios of the sampled fluids range from 2.2 to 197.8; these values are at least one order of magnitude higher than the atmospheric ratio indicating that the sampled fluids represent different degrees of mixing of air-saturated and deep sourced (crustal and magmatic) gases (Fig. 8). Stations with high ^4^He/^20^Ne ratios (185.7 and 197.9) also indicate that the collected fluids have low air contamination. However, isotopic composition of Ar in the sampled fluids show that its origin is atmospheric (Table 3), even if very little excesses of ^40^Ar has been recognized (^40^Ar/^36^Ar_samples_ (up to 309.2) > ^40^Ar/^36^Ar_air,_ i.e. 298.6^37^.

Integrated water and gas geochemistry of the sampled localities allowed to extend the geochemical database and to define regions with potential prominent fluid mixing, as well as deep or shallow fluid migration and reactions. Hydro-geochemical data, complemented by numerical simulations suggest that at some sites from the western side of the SoG we have diluted brines resulting from seawater evaporation after the Mediterranean paleo-transgression^38–40^. The depicted plumbing system is consistent with the results obtained from the samples of Group 1. Therefore, the mixing between seawater brines and freshwater can be extended for a large part of the western shore of the SoG. In contrast, the relative enrichment in fluid-mobile elements observed in samples from Group 2 (i.e., along the DSF) suggests admixing of fluids formed at higher temperatures in the subsurface (e.g.^41^ and references therein). He with Rc/Ra from 1.2 to 2.2 is within the range observed by other authors^42,43^ and interpreted as mantle-derived ^3^He along tectonic structures. This includes samples from the north western shore of the SoG, along the onshore continuation of the CT (Stations 12, 13 and 14).

He in natural fluids is mainly sourced by three reservoirs: air (^3^He/^4^He = 1.39 × 10^–6^, Ra), crust (0.01–0.03Ra^44^) and upper mantle (in this case assumed to be a Sub Continental Lithospheric Mantle (6.1Ra^45^). Following Giggenbach et al.^46^ it is possible to compute the percentage of He from these three sources by considering the He isotopic signature and the ^4^He/^20^Ne ratios of the three end members (crust and mantle ^4^He/^20^Ne > 1,000; air ^4^He/^20^Ne = 0.29). Collected fluids have a mantle derived component up to 30% (e.g. cfr. Fig. 8;^43^). This relatively high input of mantle-He, is accompanied by a high radiogenic ^4^He contribution of crustal origin, in a region with a thick sedimentary package of up to 8 km, and a Moho at ~ 25 km^47^. Stations 1–2–3, from both sides of the lake, revealed high nitrogen concentrations (up to ~ 88%) and positive δD, with very high values up to 281‰ (Hamat Tiberias, Station 2). This is, to our knowledge, the highest value ever recorded for any terrestrial gas sample. These values could be related to secondary oxidation processes^48^, which might take place either directly in the bubbling pool by microbial colonies or at shallow depth. An additional/alternative hypothesis could be the occurrence of sustained abiogenic oxidation at greater depth. This scenario is supported by (1) the above-mentioned mantle component present in the He, (2) the relatively high temperatures measured (as high as 60 °C at Hamat Tiberias, Station 2), and (3) evidence for a magmatic component in the seeping CO_2_ on the western side of the SoG (δ^13^C_CO2_ up to − 9‰). Although the measured stable isotope methane signature of i.e. δ^13^C_CH4_ = − 18.7‰ at Station 1 could indicate abiogenic origin^49^, it cannot be excluded that it is of thermogenic origin with post-genetic alteration/oxidation processes^50^. This secondary process is accompanied by isotopic fractionation leading to even more positive residual methane fractions similar to that of Station 2 (δ^13^C_CH4_ =  + 26.2‰).

Fluids from Stations 6–8 in the Hula Valley show a distinctive geochemistry. These are characterized by a He isotopic ratio (Rc/Ra) of 0.1, the lowest in the studied area, indicating that the He inventory is almost completely dominated by radiogenic ^4^He production due to decay of U and Th in the crust. This is consistent with the relatively shallow depth of the sampled well at this locality and with the CH_4_-dominated microbial gas, with possible ongoing reduction-fermentation processes at shallower levels (− 67‰ < δ ^13^C_CH4_ < − 59‰ and − 258‰ < δD_CH4_ < − 221‰).

To summarize, geochemical data suggest the migration of fluids enriched in mantle-derived volatiles and mobile elements through tectonic structures along the DSF and the CT. This is consistent with the presence of deeply-rooted structures possibly connected with magmatic intrusions at depth, or linked to the mantle-crust boundary.

### Implications for geodynamic reconstructions

Our reconstruction shows that the SoG is a key region for understanding how deformation across the DSF is presently accommodated, not only along the main N-S oriented fault system, but rather by systematic bifurcations probably inherited by early stages of the DSF emplacement. The NW–SE striking, seismically active CG Fault is the most prominent structure branching from the DSF towards the NW. It has been interpreted as a major boundary separating two distinct crustal domains, Judea-Samaria and Galilee-Lebanon^51–53^. Data collected offshore Israel and Lebanon suggest that the CG Fault terminates offshore into the NW trending normal fault scarps forming a horsetail structure^54^. This is agreement with the presence of stress partitioning along NW–SE and NNW-SSW tectonic lines. Carton et al.^54^ suggest that formation of strike-slip/transtensional features oblique to the DSF could be driven by interactions with Tethyan inherited transform faults, as suggested for other peri-Mediterranean regions^55–57^. A possible alternative is that such features could have formed during the initial stages of the DSF wrench tectonic boundary^58^. In fact, orientation of the active tectonic features recognized in the current study overlaps and fits well with that observed in the strain ellipse of sheared margins (Fig. 6).

Our data suggest at least two phases in the SoG formation: (1) an early extensional stage, driven by transtensional faulting along the DSF main track, which produced the half-graben depression deepening towards the east; (2) a post-rift < 1 Ma stage, where the DSF accommodates mainly strike-slip deformation, and trans-tensional stresses migrated northwards^9^, and eastwards along the CT. Deep-penetrating seismic images and mantle-derived He in the fluids call for the presence of deep roots on a regional scale.

### Implications for seismic hazard

It has been proposed that most of the strike-slip motion in the SoG region is currently occurring on a single fault along its eastern shore. However, analysis of historical catalogues suggests that some major earthquakes, at least the well documented AD 1837 event, strongly affected the western shore of the lake^11,31^. Moreover, the most recent swarms of seismic activity, i.e., the October 2013 and July 2018 events, were mostly localized in the NW sector, off the main DSF track.

**Figure 9:**
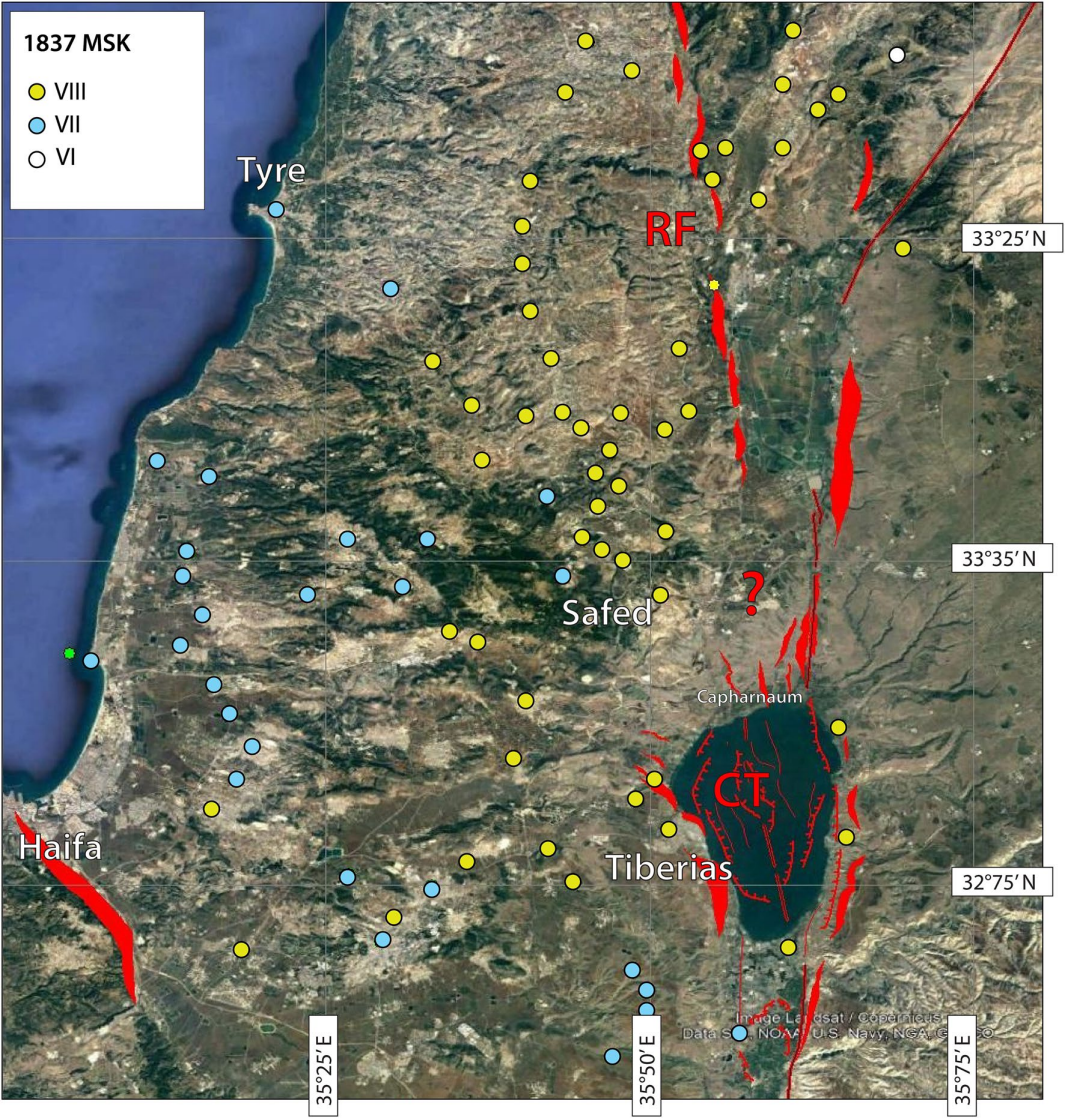
Tectonic map of the Sea of Galilee area including regional tectonic lineaments from previous studies^50–52^ and those derived from this work. Coloured circles represent intensity distribution of the AD 1837 earthquake according to^31^ (see legend for MSK intensities). We note that maximum intensities (yellow circles) align with structures located to the west of the SoG. Data available to date could not allow us to determine whether a connection between CT (Capharnaum Through) and RF (Roum Fault) could eventually exist. Satellite image is from Google Earth (https://www.google.it/intl/it/earth/; Map data: Google, Data SIO, NOAA, U.S: Navy, NGA, GEBCO, Image Landsat/Copernicus). Map compiled using the GMT package (a); image editing using Adobe Illustrator CS6.

The CT shows an orientation similar to that of the Roum Fault (Fig. 1), which extends for about 35 km from the Hula basin to the Awali River in Lebanon^59^. Activity of the Roum Fault during historical and pre-historical times was confirmed by palaeoseismic studies, which suggest at least 4–5 large seismic events with surface ruptures during the last 10 ka^59^, the latest being the AD 1837, Ms = 7.1 earthquake. Macroseismic observations^31^ indicate maximum MSK intensities distributed mainly to the west of the DSF trace, reaching up to the SoG coasts (Fig. 9). In light of these observations and in absence of onshore studies, we could then speculate that the AD 1837 rupture should have extended towards the south to include the SoG and the CT, with a fault length of over 80 km, compatible with the most reliable magnitude estimates^31^. Deformations imaged on the SoG floor by our geophysical data, are possibly the consequence of relatively recent events, such as AD 1837, since sedimentation rates ranging from 2–7 mm/year^60^ could rapidly mask strike-slip coseismic ruptures. This would also explain the lack of fault rupture evidence in the backscatter images of the lake floor. If the hypothesis of a CT-Roum Fault rupture in case of large magnitude events is reasonable, we could tentatively assume similar slip-rates (0.86–1.05 mm/year) as derived by paleoseismic analysis^59^. This estimate has deep implications for earthquake hazard assessment along the DSF system, and should be verified by further paleoseismic analyses offshore and onshore the SoG. A rate of about 1 mm/year is around one third of that estimated for the DSF north of the SoG by paleoseismic^30^ and geodetic data^61^. Therefore, the potential for large magnitude events along the CT-Roum Fault strand should be scaled accordingly.

## Summary and conclusions

Our integrated approach detected active seismogenic, deep-rooted faults in a key region along the Dead Sea continental transform. A multidisciplinary dataset reveals prominent NNW-SSE oriented transtensive deformation, forming at a left-lateral bifurcation of the sinistral Dead Sea Fault system as it enters the southern Sea of Galilee. This rhomb-shaped structural depression, marked by NW-oriented, subparallel transtensive faults, propagates from the lake’s centre towards the village of Capharnaum. We named this structure the *Capharnaum Trough*, located west of the lake’s depocenter, probably indicating a recent (< 1 Ma) major change in the strain pattern during the Sea of Galilee formation. The outgassing of mantle-derived volatiles and fluid-mobile elements through these tectonic discontinuities imply deeply rooted fluid pathways. Seismological observations and macroseismic reports of historical events suggest that structures off-track relative to the principal deformation zone could generate low to moderate magnitude seismicity, as well as large magnitude (M ≥ 7) earthquakes. In fact, the Capharnaum Trough could be considered an active segment of a larger seismogenic structure extending from the Sea of Galilee to the Lebanon restraining bend.

## Methods

The present work is based on processing and interpretation of waterborne geophysical data collected during a recent (2017) campaign, integrated with reprocessed available datasets and coupled with geochemical and seismological observations carried out in the SoG and its surroundings.

### Single-beam bathymetry

Although multibeam echosounder bathymetry was collected in the SoG^62^, we found the single-beam bathymetric data collected prior to 2004 with different methods and errors, more effective for gathering information on morphological expressions of faults. This is because they were more easily compared, mixed and homogenized, with our 2017 survey. Single-beam bathymetries were also used by Guitton and Claerbout^63^ to test algorithms for filtering non-Gaussian noise in the form of spikes inside the lake and at the track ends. The authors used triplets of irregularly spaced soundings to compile a morpho-bathymetric map of the SoG, which was found to be a very sensitive tool for imaging ancient shorelines and potential archaeological sites^63^. Such data were resampled for the present study, and their positioning verified using as a reference our new survey (see below), and subsequently gridded with the GMT nearest-neighbour algorithm^64^. In this way, a new NetCDF grid of data was obtained and displayed as a colour topographic gradient map, subsequently used as a base for mapping active faults (Supplementary Fig. S1).

### High-resolution seismic reflection lines

A grid of high-resolution chirp sonar profiles was collected in the SoG as part of a large interdisciplinary project aimed at examining fluid pathways and seismicity^1^. These data were acquired using a *Datasonics Chirp-III* system, generating a frequency-modulated sweep of acoustic signals ranging from 2 to 7 kHz. Data were processed and interpreted using the *SeisPrho* software package^65^. An additional ~ 57 km of single channel seismic data collected during surveys carried out from 1983 to 2004 was examined to improve fault mapping (Fig. 4). An example of high-resolution single-channel seismic reflection line is displayed in Supplementary Fig. S2.

### Side-scan sonar imaging

Side-scan sonar images were collected during the 2017 survey using a *Starfish 450 F* system, towed in a fixed position aside the boat. Data were collected using the Scanline interface software, exported in XTF format and processed with *Caris Hips&Sips* software to obtain a 25 cm resolution mosaic. Scan width was 100 m for each side.

### Multichannel seismic lines

Deep-penetrating seismic images below the SoG were obtained using a grid of 20 multichannel seismic reflection profiles (total length 180 km) collected in 1997 stored in SEG-D format. The seismic source was a single 400 in^3^ water gun, operating at a pressure of 1,820 psi. The receiver was a 600-m-long, 48-channel streamer with a 12.5 m group-interval. Shot interval was 12.5 m, sampling rate 2 ms. and record length 4 s. Seismic data were reprocessed for the purpose of our work using an industrial package (Disco/Focus) by Paradigm Geophysical, following a non-standard sequence (Supplementary Fig. S3), with the aim of obtaining depth-migrated sections. Pre-processing included: (1) swell noise removal by time-variant band limited noise suppression. Data were decomposed into good and bad signal frequency bands, with signals up to 18 Hz included within the noise frequency band. The noise and signal envelopes were smoothed by a window of 60 ms. Noise suppression was performed by comparison of noise envelope with signal envelope with a threshold level of 0.2 times the signal envelope; (2) setting acquisition geometry; (3) interactive noisy trace editing; and (4) spherical divergence correction. Processing steps included: (5) water column muting; (6) predictive deconvolution with operator length of 255 ms, prediction lag of 4 ms and pre-whitening of 0.2%. and filter design window of 0–2 s. and an application window of 0–4 s; (7) DC-bias removal and time variant trace amplitude equalization; (8) time variant band-pass filtering with a frequency band of 4/8–72/96 Hz; (9) CDP sorting and velocity analysis; (10) random and coherent noise reduction by f-k and tau-p velocity filtering in the shot, receiver and common midpoint domains; (11) bottom surface multiple removal using 2D surface related multiple suppression (SRME) technique and adaptive filters; (12) normal move out (NMO) and dip move out (DMO) corrections; (13) NMO removal and velocity analysis; (14) NMO and stacking; and (15) finite difference time and depth migration after iteratively smoothing and refining the velocity model. Uninterpreted depth/time migrated seismic lines KIN_05 and KIN_07 highlight processing results (Supplementary Figs. S4, S5a and S6a). In order to determine correctly active fault traces, i.e., traces of faults showing an expression as close as possible to the surface, instantaneous attributes (reflection strength) of the time/depth migrated seismic images (Supplementary Figs. S5b and S6b) were analysed in conjunction with filtered near-offset sections (Supplementary Fig. S7), more sensitive to the shallow geometries of the faults. This approach preserved the high frequencies of the seismic signal and the full resolution of the data close to the lake floor, allowing better recognition of fault ruptures within the sedimentary sequence. Fault mapping was performed using also near-offset sections (Supplementary Fig. S7). Processing of the near-offset sections included time-variant band-pass filtering and a spike deconvolution, both performed using the SeisPrho software package^65^.

### Seismology

A local network of 17 seismic stations (Fig. 1 for locations) was deployed around the SoG from September 2017 to August 2018, as part of an international project on local seismicity^1^. The network was composed of 12 3-components short period *Lennartz LE-3Dlite* 1 Hz sensors, equipped with *Datacube Omnirecs* digitizers powered by a local electrical source or solar panels, and operated continuously for 10 months except for a few gaps. We applied a STA/LTA routine to the seismic sequence, which resulted in 789 detections, then manually picked 14,263 P- and 10,862 S-wave phases for 666 events located with the regional velocity model for Israel of Aldersons et al.^66^. We used the *automag* routine implemented in the *SeisAn* package^67^ for magnitude estimation, and *FPFit*^68^ integrated in the package to assess focal mechanisms using P-wave first motion polarities and depth.

### Geochemistry of tectonic-related fluid seepage

Fluids were collected from natural springs around the SoG, at wells in the Hula Valley and at natural springs along the DSF (Fig. 1). Gas was sampled at bubbling seeps using a plastic funnel positioned upside-down and connected by silicone tubes to brine-filled glass bottles, subsequently hermetically sealed, or collected in two valve glass samplers. Prior to sampling, the sampling system was flushed for ~ 10 min to reduce the potential air contamination of the collected sample. In absence of bubbling, water was collected in crimped 245 ml glass flasks for dissolved gas analyses. Dissolved gas samples were prepared at GEOMAR (Germany) extracting the gas phase from the collected waters (245 ml vials) by replacing 10 ml of water with He-headspace (HS). The samples were then left for 1 h-equilibration at room temperature. HS-gas concentrations and gas composition of the “two valve glass samplers” were determined with a *Shimadzu GC2014* gas chromatograph equipped with *TCD, FID* detectors (column: *HaysepTM* Q80/100, 2 m, 1/8′’; carrier gas: He). The detection limit for hydrocarbons was about 0.1 ppmV and 100 ppmV for permanent gases. Precision of measurements was about 4% (2SD). Stable carbon isotope ratios (^13^C/^12^C) of methane, ethane, and carbon dioxide were measured by using a continuous flow isotope ratio mass spectrometer (*Thermo MAT253*) at GEOMAR. Prior oxidation of methane and subsequently ethane in a 1,150 °C furnace gases, had been separated from CO_2_ in a coupled *Thermo Trace GC* (carrier gas: He; packed column: *ShinCarbon*, 1.5 m). Separated CO_2_ bypassing the hot oven was measured by the mass spectrometer at different retention times. Stable isotope ratios are reported in the δ-notation with respect to *Vienna Pee Dee Belemnite* (VPDB). Analytical precision of the reported isotopic composition is ± 0.3‰.

He and Ne isotopic composition was determined at the National Institute of Geophysics and Volcanology (INGV), Italy. ^3^He, ^4^He and ^20^Ne were determined by injecting He into a split flight tube mass spectrometer (model *GVI-Helix SFT*) and Ne into a multi-collector mass spectrometer (model *Thermo-Helix MC plus*), after standard purification procedures^69^. The results have an analytical error generally less than 1%. The measured He isotopes values are reported as R/Ra (where R is the ^3^He/^4^He in the sample, Ra is the ^3^He/^4^He ratio of air, i.e. 1.4 × 10–6). The R/Ra values were also corrected for atmospheric contamination by using the approach in Giggenbach et al.^46^ and these are expressed as Rc/Ra. Ar-isotope composition was measured in a multi-collector mass spectrometer (*MC-GV Instruments*), with analytical uncertainty of 0.5%.

Water samples were collected at each locality with triplicates in 14 ml plastic vials (i.e. non-filtered, 0.2 µm filtered, filtered and HNO_3_ acidified). Elemental analyses were carried out at GEOMAR (Germany) using ion chromatography (*IC, METROHM* 761 Compact) and inductively coupled plasma optical emission spectrometry (*ICP-OES, VARIAN 720-ES*). The results are reported in Table 1 in millimoles per litre (mM) or micromoles per litre (µM). More detailed descriptions of the methods used are provided at https://www.geomar.de/en/research/fb2/fb2-mg/benthic-biogeochemistry/mg-analytik/ or previous publications (e.g.^41,70,71^).

## Supplementary information

Supplementary file1 (PDF 2975 kb)

## Data Availability

All data used for tectonic reconstruction, including waterborne geophysical recordings and geochemical analysis will be available at the data repository of ISMAR-CNR (https://www.ismar.cnr.it/products/data-sharing).
