# Supplementary material for: Neotectonics of the Sea of Galilee (northeast Israel): implication for geodynamics and seismicity along the Dead Sea Fault system
Source: Sci Rep. 2020 Jul 20;10:11932. doi: 10.1038/s41598-020-67930-6 (PMC7371699; doi:10.1038/s41598-020-67930-6)
Supplement: Supplementary file 1 — Supplementary file1 (PDF 2975 kb) [file 41598_2020_67930_MOESM1_ESM.pdf]

Supplement Information to

Neotectonics of the Sea of Galilee (northeast Israel): implication for geodynamics and seismicity along the Dead Sea Fault system

Luca Gasperini[1], Michael Lazar[2], Adriano Mazzini[3], Matteo Lupi[4], Antoine Haddad[4], Christian Hensen[5], Mark Schmidt[5], Antonio Caracausi[6], Marco Ligi[1], Alina Polonia[1]

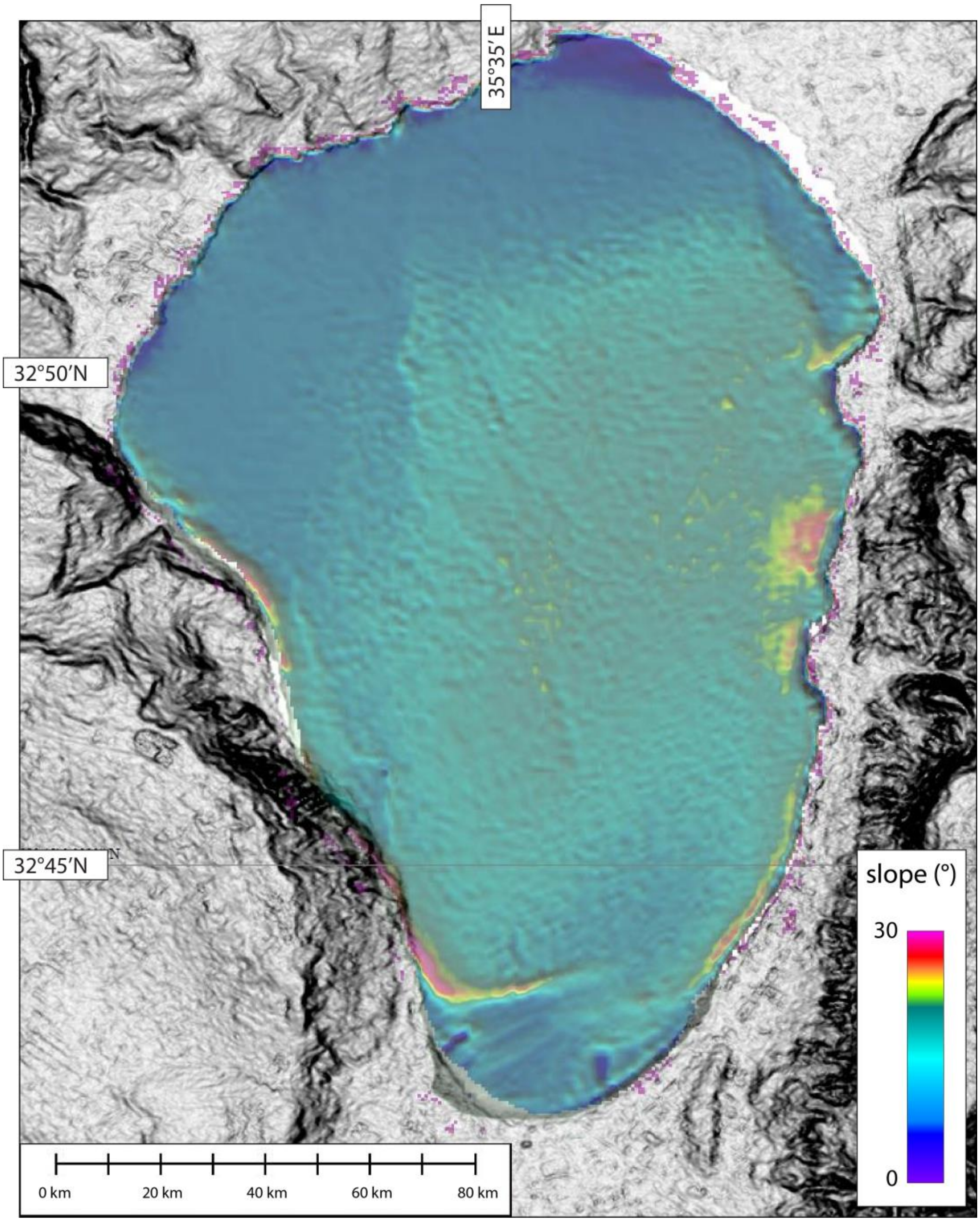

Supplementary Figure S1. *Slope map of the SoG obtained compiling data from Guitton and Claerbout, (2004). Topography onshore is from https://search.earthdata.nasa.gov/search/. Map was compiled using the GMT package (https://www.generic-mapping-tools.org/), while image editing was carried out using Adobe Illustrator CS6.*

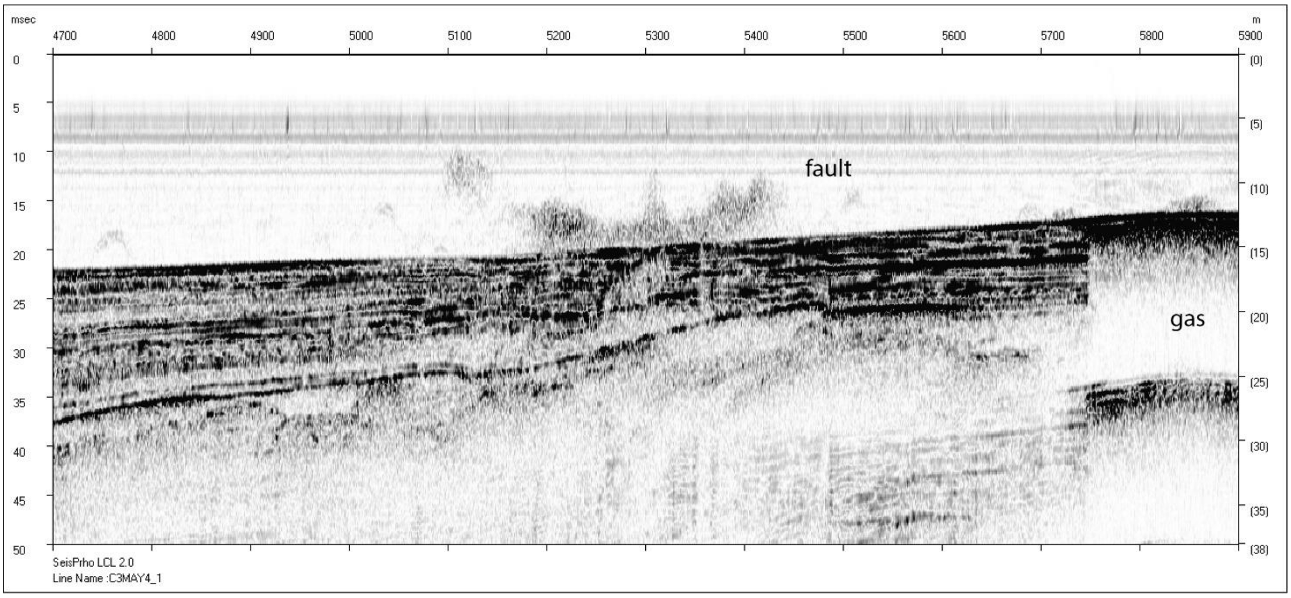

Supplementary Figure S2. *Example of sub-bottom chirp profile from the NW Sea of Galilee. Where gas is present (to the right of the section), it is not possible penetrating the sedimentary sequence; without gas, the seismic profiles image a relatively thick sequence of plane parallel beds locally cut by faults.*

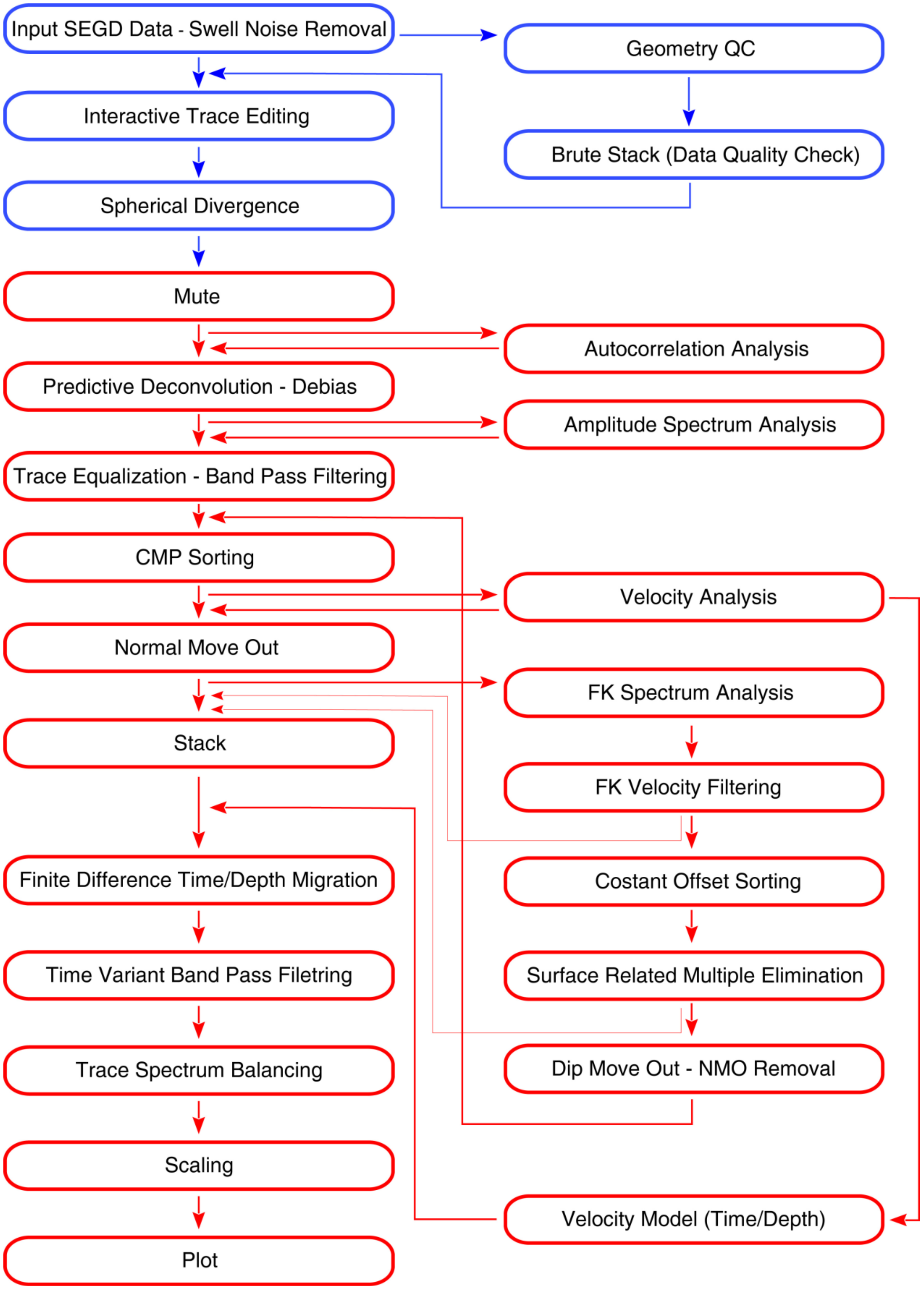

Supplementary Figure S3. *Flow chart describing the processing sequence applied to MCS data. Blue and red blocks highlight the pre-processing and processing flows, respectively (editing Adobe Illustrator CS6).*

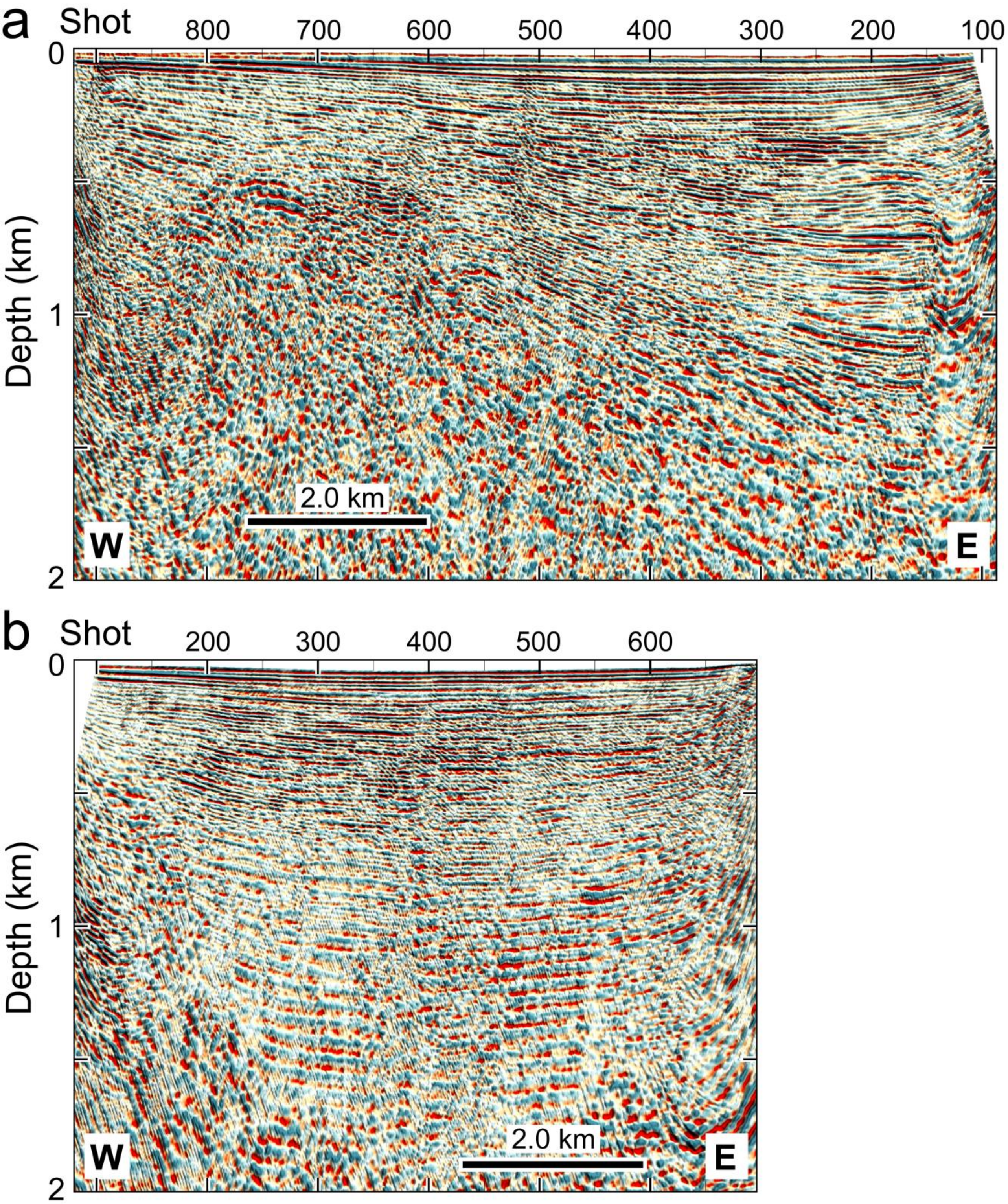

Supplementary Figure S4. *Finite difference depth migration results. During processing, particular care was devoted to the removal of water layer reverberations affecting strongly the entire seismic record and masking reflections below the first multiple arrival, as evidenced in the brute stack section shown in Supplementary Fig. S7b.* ***(a)*** *Depth migrated profile KIN_05 and* ***(b)*** *KIN_07. Final image editing was carried out using Adobe Illustrator CS6.*

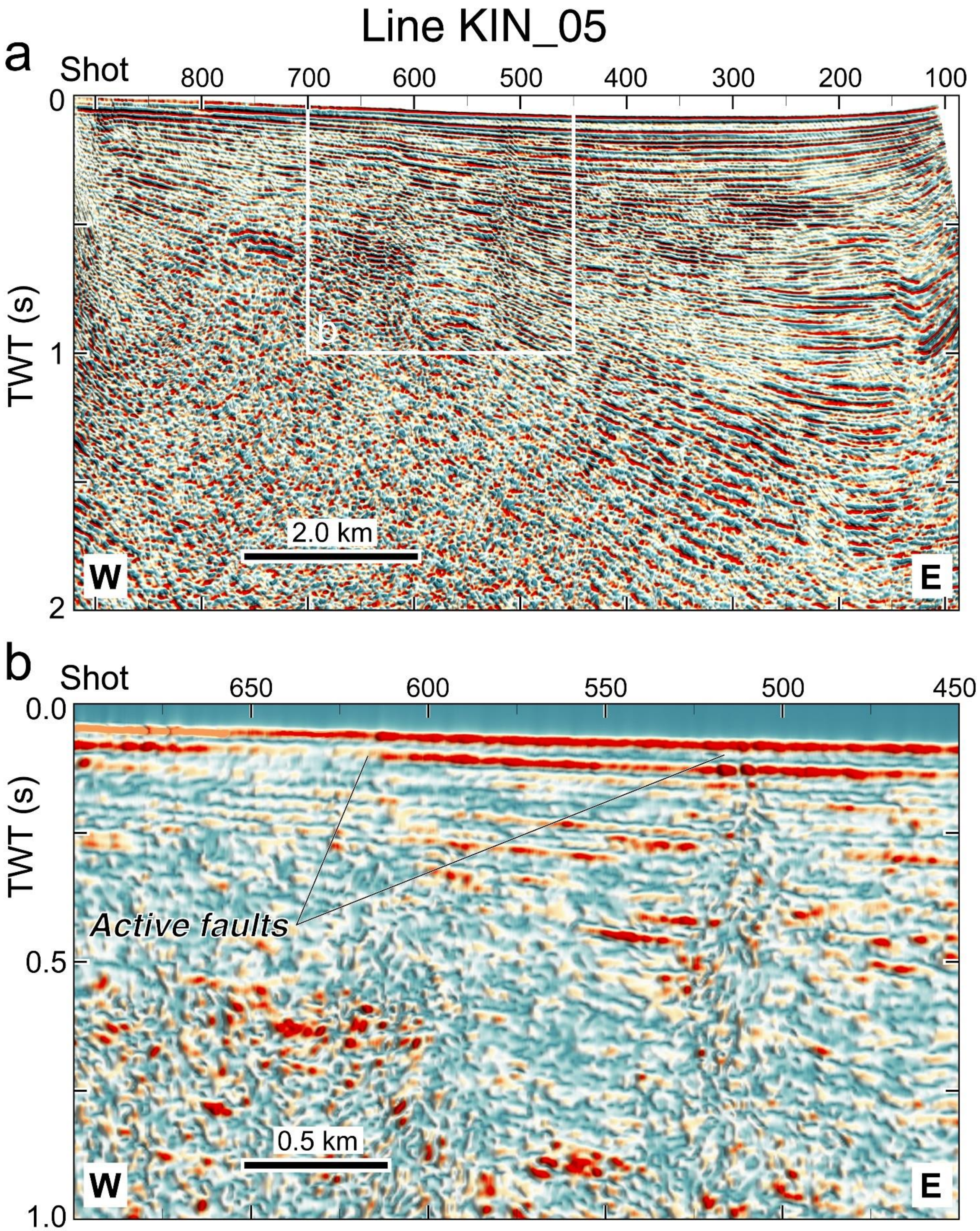

Supplementary Figure S5. *Active faults cutting seismic line KIN_05.* ***(a)*** *Finite difference time migration. White box indicates complex seismic attribute details shown in* ***b****.* ***(b)*** *Instantaneous amplitude (envelope) provides information on reflection strength and lateral continuity of seismic reflectors. Final image editing was carried out using Adobe Illustrator CS6.*

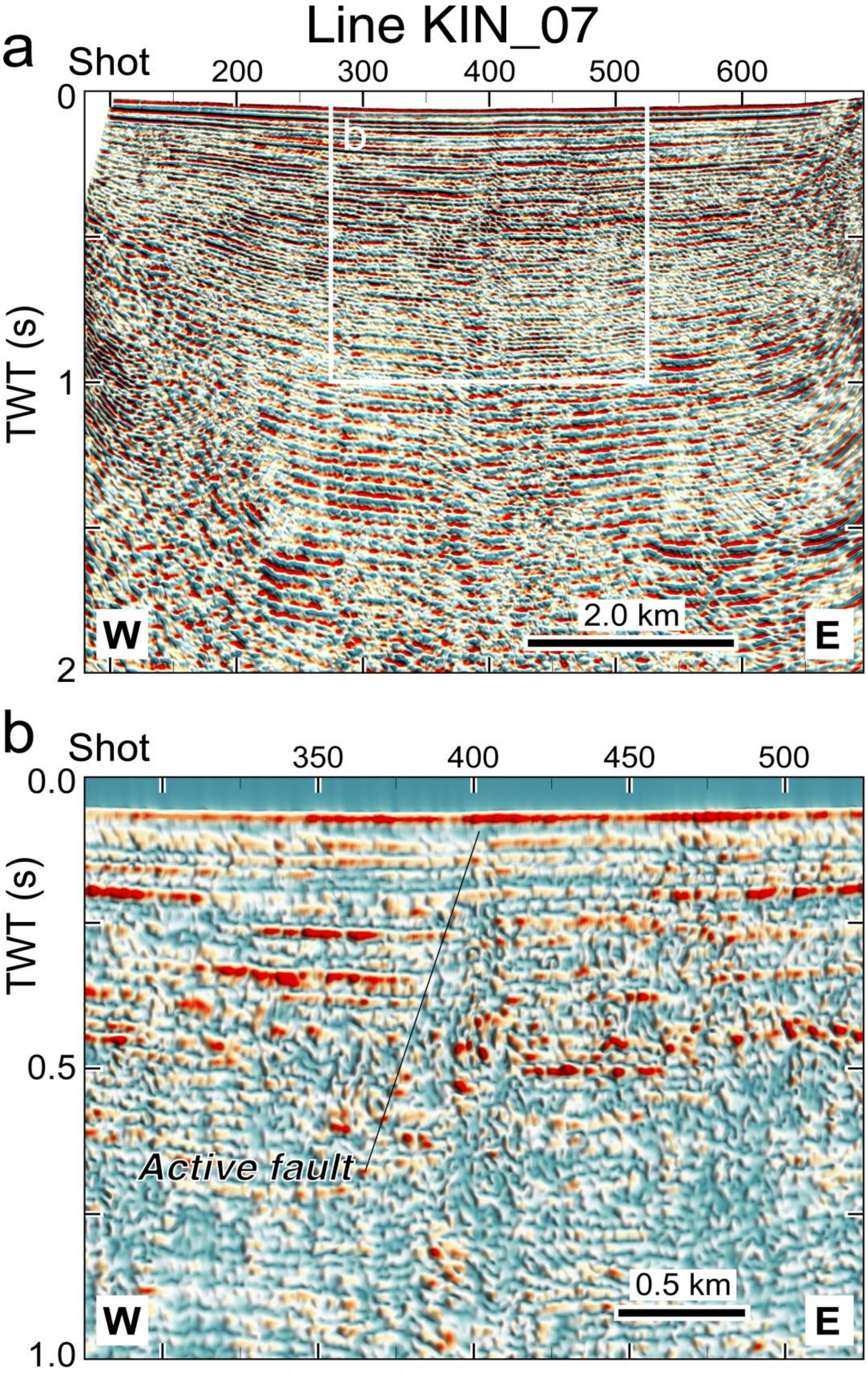

Supplementary Figure S6. *Active faults cutting seismic line KIN_07.* ***(a)*** *Finite difference time migration. White box indicates complex seismic attribute details shown in* ***b****.* ***(b)*** *Instantaneous amplitude. Final image editing was carried out using Adobe Illustrator CS6.*

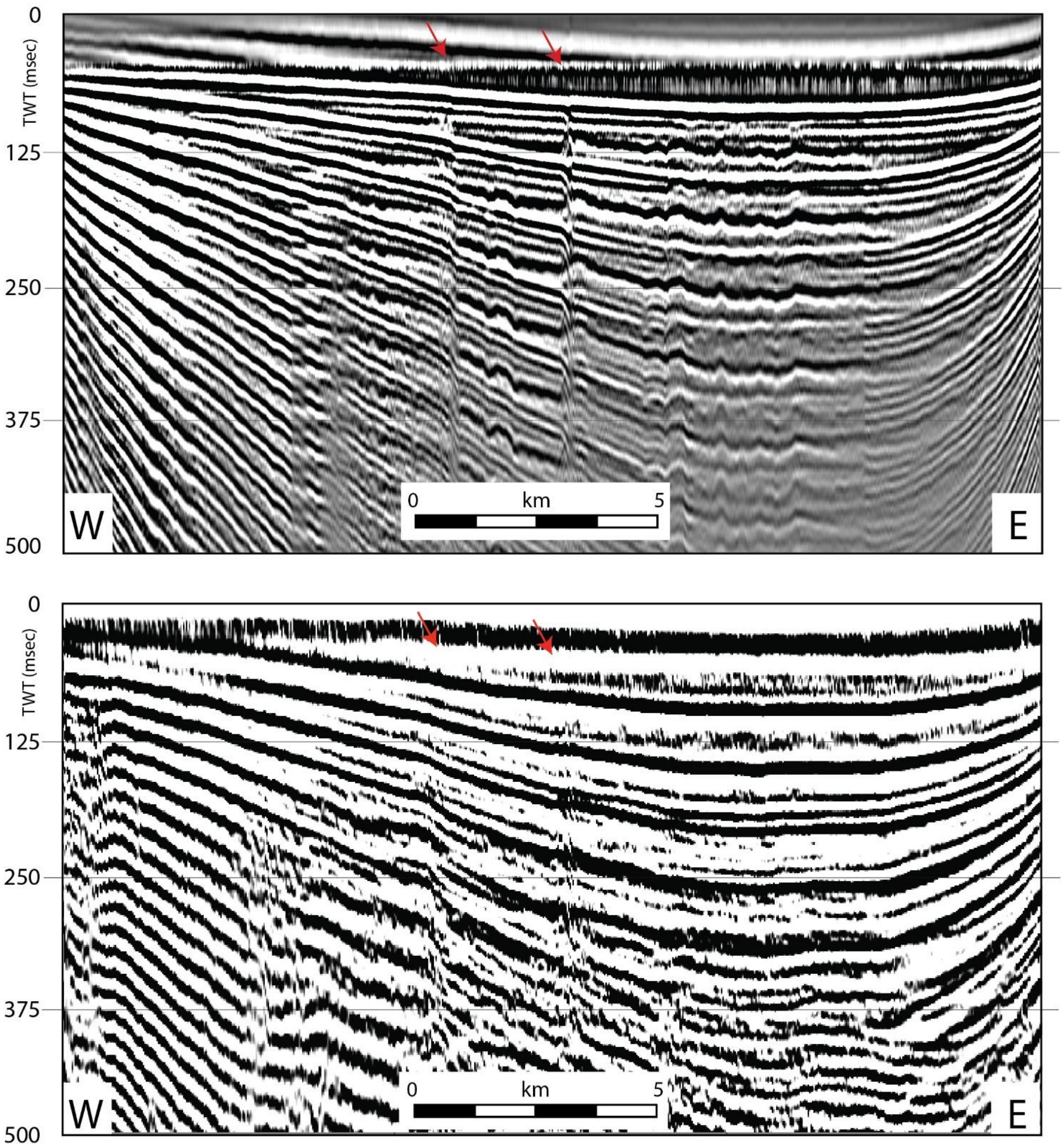

Supplementary Figure S7. *Near-offset section (top) and brute stack for data quality control (bottom) of seismic profile KIN_05. Depth and time migrated seismic images were used together with filtered near-offset and instantaneous attributes sections for more accurate fault picking. Final image editing was carried out using Adobe Illustrator CS6.*
